# Proteomics for prediction of disease progression and response to therapy in diabetic kidney disease

**DOI:** 10.1007/s00125-016-4001-9

**Published:** 2016-06-25

**Authors:** Michelle J. Pena, Harald Mischak, Hiddo J. L. Heerspink

**Affiliations:** 1 Department of Clinical Pharmacy and Pharmacology, University of Groningen, University Medical Center Groningen, P.O. Box 30.001, 9700 RB Groningen, the Netherlands; 2 BHF Glasgow Cardiovascular Research Centre, University of Glasgow, Glasgow, UK; 3 Mosaiques Diagnostics GmbH, Hannover, Germany

**Keywords:** Diabetes mellitus, Kidney disease, Proteomics, Review

## Abstract

The past decade has resulted in multiple new findings of potential proteomic biomarkers of diabetic kidney disease (DKD). Many of these biomarkers reflect an important role in the (patho)physiology and biological processes of DKD. Situations in which proteomics could be applied in clinical practice include the identification of individuals at risk of progressive kidney disease and those who would respond well to treatment, in order to tailor therapy for those at highest risk. However, while many proteomic biomarkers have been discovered, and even found to be predictive, most lack rigorous external validation in sufficiently powered studies with renal endpoints. Moreover, studies assessing short-term changes in the proteome for therapy-monitoring purposes are lacking. Collaborations between academia and industry and enhanced interactions with regulatory agencies are needed to design new, sufficiently powered studies to implement proteomics in clinical practice.

## Introduction

Diabetic kidney disease (DKD), or diabetic nephropathy, is associated with a high risk of cardiovascular disease and progressive loss of renal function. The presence of reduced kidney function in patients with type 2 diabetes predominantly accounts for the observed increase in mortality [1]. The increase in the prevalence of diabetes mellitus is projected to lead to an increase in the prevalence of DKD and the incidence of renal replacement therapy (RRT). Indeed, diabetes is the leading cause of end-stage renal disease (ESRD), and the number of prevalent cases of ESRD in the USA continues to rise by about 21,000 per year [2]. Moreover, the incidence of RRT because of type 2 diabetes is about sixfold higher than that because of glomerulonephritis, and approximately 20-fold higher than that because of cystic kidney disease [2]. In Europe, the prevalence rate of RRT in most countries has also grown in the past decade [3]. It has been forecast that by 2030 the worldwide number of patients undergoing RRT will have doubled [4].

Diagnosis of DKD is based on the detection of albuminuria and a progressive decline in estimated glomerular filtration rate (eGFR) [5]. Reduced eGFR is the consequence of compromised kidney function and the substantial loss and destruction of the glomeruli. Increased albuminuria is often the first clinical indicator of the presence of DKD and is the strongest tool for prognosis and monitoring response to therapy [6–8]. The terms microalbuminuria (urinary albumin excretion [UAE] 30–300 mg/day, or 20–200 μg/min, or 30–300 mg/g creatinine) and macroalbuminuria (UAE >300 mg/day, or >200 μg/min, or >300 mg/g creatinine) are clinically used to indicate the severity of albuminuria [9, 10]. Clinical practice guidelines today advocate regular assessment of albuminuria and eGFR to monitor an individual’s risk of cardiovascular disease and ESRD.

The cornerstone of treatment for DKD consists of tight control of blood glucose and blood pressure, preferably with drugs that target the renin–angiotensin–aldosterone system (RAAS). Currently, the available therapies are usually initiated at more advanced stages of DKD, characterised by clinically evident manifestations of elevated arterial blood pressure, increased albuminuria and/or low eGFR [11]. A recently published simulation study combining clinical trials of patients with type 2 diabetes at early, mid- and advanced stages of DKD demonstrated that RAAS intervention in the earliest stages of disease was most beneficial in delaying ESRD, and that this treatment effect was even more pronounced among younger patients [12]. The simulation showed that ESRD was markedly delayed among patients with an initial response in albuminuria, whereas non-responders showed only a small benefit compared with placebo [12]. These results highlight the importance of early treatment initiation in diabetes. Furthermore, novel strategies are needed to identify which patients at risk of kidney disease would benefit most from early treatment. It is a clinical challenge to identify patients at high risk while their eGFR and albuminuria are still in the normal range.

**Fig. 1:**
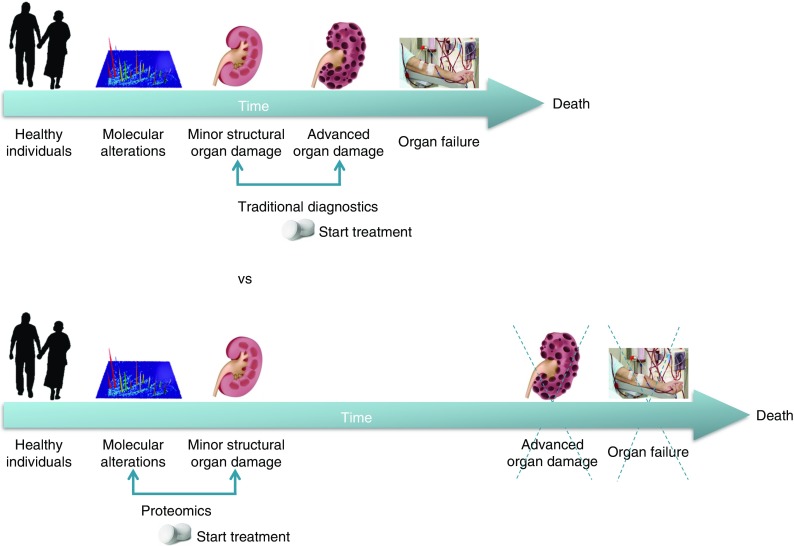
Early identification with proteomics of patients at risk of kidney disease, prior to organ damage, and initiation of appropriate treatment is a strategy to interrupt disease progression to ESRD and death

The data currently available indicate that early intervention, prior to organ damage detectable by albuminuria and/or reduced eGFR, would be the best preventative treatment (Fig. 1). However, evidence from randomised placebo-controlled trials addressing the relevant hard endpoints—ESRD, doubling of serum creatinine or halving of kidney function—to support these results is lacking, since such a trial would require a very large population and a very long follow-up. To study the efficacy of early intervention, a change in endpoint is required. Current endpoints to determine drug efficacy are ESRD or doubling of serum creatinine. These endpoints by definition are late events in the progression of DKD. Alternative endpoints that take a shorter time to manifest, such as a 30% decline in eGFR or a transition in albuminuria stage, have been proposed [13, 14]. A new European Medicines Agency guideline is currently under consideration in which alternative endpoints are proposed such as the occurrence of stage 3 chronic kidney disease (CKD) or prevention or reduction of albuminuria [15]. General acceptance of these endpoints would open a path towards early intervention in DKD.

Novel biomarkers are one strategy to improve identification of kidney disease at its early stages and to tailor therapy for those at highest risk. In addition, they can help the understanding of the aetiology of kidney disease progression and provide insight into novel therapeutic targets. In the last decade, many biomarkers have been discovered to be associated with DKD. Many of these biomarkers are proteins, reflecting the important role of this group of molecules in the (patho)physiology of DKD. Proteins in blood could represent peripheral pathophysiological processes such as inflammation, e.g. tumour necrosis factor receptor (TNFR) 1 and TNFR2 [16, 17]. Proteins in urine could reflect local processes in the kidneys that may be sensitive to alternations in kidney physiology. Since several pathways are probably involved in DKD progression, no single biomarker has yet replaced albuminuria to predict renal risk. Instead, a panel of multiple protein biomarkers capturing the different pathophysiological pathways of DKD may be more likely to reliably and accurately predict kidney disease progression or ESRD [18]. These panels may consist of proteins or peptides identified through hypothesis-driven studies of known proteins involved in the pathophysiology of DKD or developed through hypothesis-free high-throughput approaches.

In this review we describe the recent literature on biomarker panels in DKD, both with respect to disease progression and to response to treatment. We also discuss individual biomarkers and place them in the context of their (patho)physiological role in DKD.

## Multiple protein biomarkers for prediction of disease progression

### Hypothesis-driven protein panels

Many biomarker studies in patients with DKD have been performed and many different clinical and novel proteins have been proposed as valuable indicators or predictors of kidney disease [16, 17, 19–30]. These studies often focus on one specific mechanism of disease, such as inflammation, fibrosis or tubular damage, highlighting the relevance of single disease mechanisms and providing important insight into the disease aetiology. The heterogeneity of diabetes and DKD is well recognised [31], and simultaneous measurement of several biomarkers has been shown to improve risk stratification [32]. However, only a few studies have been conducted that assessed the predictive performance of hypothesis-driven protein biomarker panels. Recent studies on multiple biomarkers for prediction of kidney disease progression in diabetes are summarised in Table 1.

**Table 1:** Overview of selected hypothesis-driven studies investigating the predictive value of biomarker panels for DKD progression

Study	Design	Patient number and diabetes type	Duration of follow-up, years	Biofluid	Endpoint	Candidate biomarkers	Pathophysiological domains represented by biomarkers	External validation
Persson et al (2008) [33]	Post hoc analysis of randomised controlled trial	269 T2DM	2	Plasma	Onset of diabetic nephropathy	hs-CRP, IL-6, fibrinogen, von Willebrand factor, sVCAM-1, sICAM-1, sE-selectin, TGF-β, AGE peptides	Inflammation, endothelial dysfunction	No
Astrup et al (2008) [34]	Observational cohort	199 + 192 T1DM	10	Plasma	Mortality and GFR decline	CRP, IL-6, sICAM-1, secreted phospholipase A2, sVCAM-1, PAI-1, sICAM-1	Inflammation, endothelial dysfunction	No
Verhave et al (2013) [35]	Observational cohort	83 T1DM + T2DM	2.1	Urine	Overt diabetic nephropathy	MCP-1, TGF-β1	Inflammation, fibrosis	No
Agarwal et al (2014) [36]	Case–control study	67 + 20 T2DM	2–6	Urine/plasma	eGFR decline, progression to ESRD, and/or death	Urinary and plasma C-terminal, FGF-23, plasma VEGF-A	Inflammation, fibrosis, angiogenesis, glomerular injury, mineral metabolism, tubulointerstitial injury	No
Pena et al (2015) [37]	Observational cohort	82 T2DM	4	Serum	eGFR decline	MMPs, tyrosine kinase, podocin, CTGF, TNFR1 sclerostin, MCP-1, YKL-40, NT-proCNP	Inflammation, fibrosis, angiogenesis, endothelial dysfunction, mineral metabolism, lipid metabolism, glomerular damage	No

Because of inconsistencies in the methodology used to assess the performance of the biomarker panels (e.g. testing the biomarker panels on top of clinical predictors [albuminuria and/or eGFR, etc.] vs testing the biomarker panels without clinical predictors), we do not report performance measures (e.g. AUC for receiver operating characteristic)

AGE peptides, advanced glycation end-product peptides; CRP, C-reactive protein; CTGF, connective tissue growth factor; FGF, fibroblast growth factor; GFR, glomerular filtration rate; hs-CRP, high-sensitivity C-reactive protein; MCP-1, monocyte chemoattractant protein-1; MMPs, matrix metalloproteinases; NT-proCNP, N-terminal fragment of C-type natriuretic peptide precursor; PAI-1, plasminogen activator inhibitor-1; sE-selectin, soluble E-selectin; sICAM-1, soluble intercellular adhesion molecule-1; sVCAM-1, soluble vascular adhesion molecule-1; T1DM, type 1 diabetes; T2DM, type 2 diabetes; VEGF-A, vascular endothelial growth factor-A; YKL-40, Chitinase 3-like 1 protein

In a post hoc analysis from the Irbesartan Microalbuminuria Study-2 (IRMA-2), it was shown that a panel of biomarkers reflecting endothelial dysfunction and inflammation predicted progression to diabetic nephropathy over 2 years in 269 patients with type 2 diabetes and microalbuminuria. The predictive capacity of these endothelial biomarkers was independent of traditional risk markers [33]. Another study conducted in 199 patients with type 1 diabetes and diabetic nephropathy showed that markers of endothelial dysfunction and inflammation could predict all-cause mortality and cardiovascular disease after 10 years of follow-up [34]. Further support for the role of inflammation in early kidney disease progression comes from an observational cohort of 81 patients with type 1 or type 2 diabetes, followed for a median of 2.1 years, which demonstrated that a panel of multiple urinary cytokines predicted rapid renal functional decline in diabetic nephropathy [35]. In an observational study of 67 US veterans with CKD and 20 age-matched healthy controls followed for 2–6 years, fibroblast growth factor (FGF)-23 and vascular endothelial growth factor (VEGF)-A predicted disease progression independently of albuminuria [36]. The importance of multiple proteins representing multiple pathways of kidney disease progression was corroborated by a multiple biomarker study. In an observational study of 82 patients with type 2 diabetes followed for 4 years, a panel of 13 novel proteins was associated with accelerated renal functional decline beyond established risk markers [37]. Markers of inflammation, fibrosis, angiogenesis and endothelial function were identified.

One common denominator in the above-mentioned studies is the presence of proteins related to endothelial dysfunction in all biomarker panels. Endothelial dysfunction is considered an initial step of the atherosclerotic process, because diabetes substantially impairs vasodilating properties of the endothelium, leading to impaired vasodilation and ultimately endothelial dysfunction [38]. In addition, the glomerular endothelium represents the first part of the glomerular barrier that interacts with the flowing blood. Endothelial dysfunction may thus be considered an early sign of glomerular damage. The endothelium is covered by a polysaccharide protein gel-like structure called the glycocalyx. Through its negative charge the glycocalyx prevents leakage of albumin, which is also negatively charged, through the vessel wall [39]. In addition, the glycocalyx plays an important role in vascular remodelling by binding local growth factors such as VEGF and FGF. Loss of the glycocalyx has been described in patients with diabetes [40], where exposure to high glucose and lipids activates glycocalyx-degrading enzymes such as heparanase [41]. This facilitates the development of vessel wall injury, albumin leakage and recruitment of inflammatory initiators, which ultimately culminates in renal damage. This pathophysiological framework may explain the clinical association of albuminuria with kidney disease and, possibly also more generally, with vascular disease progression [39].

## Proteomics of disease progression

### Hypothesis-free proteomic panels

The past decade has yielded a number of hypothesis-free biomarker studies using proteomic approaches. High-throughput profiling of the proteome permits the assessment of components of proteins within a biological sample. The proteome consists of all the protein products derived from an individual’s full genetic code. It is estimated that more than 500,000 proteins comprise the human proteome, derived from ∼35,000 genes in the human genome [42, 43]. Biological samples such as urine, plasma, serum or tissue can be systematically analysed, with the goal of identifying, quantifying and discerning the function of all observable proteins in health and disease.

Theoretically, proteomics appears an ideal tool to study molecular mechanisms, as it bridges the gap between what is encoded in the genome and its translation into proteins. Early proteomic studies using surface-enhanced laser desorption ionisation (SELDI)-based approaches have led to some interesting discoveries [44, 45], and the past 10 years of proteomic studies in diabetic nephropathy have increased our knowledge of the molecular mechanisms involved in its pathogenesis [46]. However, proteomics still faces multiple challenges, among them the wide dynamic range of the proteome (spanning over ten orders of magnitude), protein modifications interfering with analysis, a substantial error rate in the experimental data, and the inability to amplify proteins (in contrast to nucleic acids). In addition, the proteome is highly variable, which further increases the need to analyse a large number of samples to obtain significant results [47]. Proteomics typically relies on the use of mass spectrometers to assess proteins and peptides [48]. A specialised discipline within proteomics, the so-called peptidomic approach, studies protein fragments/peptides that are generated in vivo [49–51]. Peptidomics is a feature-based method, where mass spectrometry (MS) data on large numbers of clinical samples are collected and compared. These discriminatory MS features can be tabulated, used as a diagnostic tool, and, for purposes of understanding molecular mechanisms, undergo tandem MS experiments to gain information on biological identity. These peptides represent the functional output of a cell or organ, and thus reflect the ‘biological status’ of an organism.

The use of high-throughput profiling techniques allows the generation of a single score that integrates multiple peptides. Untargeted proteomics aims to simultaneously assess hundreds of peptides, and thus strongly supports the generation of such multidimensional scores. Recent years have seen the development of both plasma and urine proteomic scores.

### Urinary proteomics

The measurement of proteins in urine has been used for many centuries to diagnose kidney disease [52]. Often referred to as a ‘liquid biopsy’ [53], under physiological conditions urine is generated in the kidney and about 70% of urinary proteins and peptides are derived from the kidney [54]. Urine has been a preferred target for peptidomic approaches, since it contains large quantities of multiple peptides. In addition, it is conceivable that many of the urinary peptides are associated with kidney pathophysiology and can provide information about the onset and progression of DKD. Urinary peptidomics is therefore viewed as a platform to discover biomarkers of kidney disease. Additionally, urine has the advantage of being easy to collect, though analysis does require normalisation to account for differences in urinary output. Urinary proteomics and peptidomics have gained much attention as a tool for the identification of diagnostic and prognostic biomarkers of kidney diseases [55], and may represent an important step forwards in the non-invasive diagnosis of kidney disease. Indeed, studies on urinary proteomics and DKD have been widely published and reviewed [18, 32, 56, 57].

A number of candidate urinary proteomic biomarkers have been identified that can predict kidney disease progression in diabetes (Table 2). In an early study of urinary proteomics in Pima Indians, proteomic profiles were able to predict macroalbuminuria 10 years prior to the development of nephropathy [58]. Merchant et al identified three peptides that decreased (fragments of type IV and type V α1 collagens and tenascin-X) and three peptides that increased (fragments of inositol-pentakisphosphate 2-kinase, zona occludens 3 and FAT tumour suppressor 2) in the urine of patients with type 1 diabetes and early renal functional decline [49]. In a study in type 1 diabetes (*n* = 465), a panel of four protein biomarkers (Tamm–Horsfall glycoprotein, α1-acid glycoprotein, clusterin and progranulin) predicted early renal damage [59]. When coupled with data from kidney biopsies, the results indicated that urinary peptide fragments reflect changes in expression of intact proteins in the kidney [59]. Additional evidence of urinary proteomics’ ability to predict renal functional decline was recently provided, when urinary haptoglobin was shown to be able to predict early renal functional decline in patients with type 2 diabetes [60]. In a cross-sectional study comparing CKD patients with varying underlying aetiologies of disease with healthy controls, 273 peptides (later known as the CKD273 score) were identified as being associated with CKD [50].

**Table 2:** Overview of selected hypothesis-free studies investigating the predictive value of biomarker panels for DKD progression

Study	Design	Patient number and diabetes type	Duration of follow-up, years	Biofluid	Endpoint	Platform	External validation
Otu et al (2007) [58]	Nested case–control	31 + 31 T2DM	10	Urine	Development of diabetic nephropathy	SELDI-TOF-MS	Cohort separated into training and validation sets
Merchant et al (2009) [49]	Case–control	21 + 40 T1DM	10–12	Urine	Progressive early renal functional decline	LC-MALDI-TOF-MS	No
Schlatzer et al (2012) [59]	Observational cohort	652 T1DM	6	Urine	Development of micro- or macroalbuminuria and/or early renal functional decline	LC-MS/MS	Validation of discovered peptides by ELISA
Zürbig et al (2012) [61]	Observational cohort	35 T1DM + T2DM	10–15	Urine	Development of macroalbuminuria	CE-MS	Yes
Bringans et al (2012) [78]	Observational cohort	279 T1DM + T2DM	4	Plasma	Renal functional decline	iTRAQ-MS	No
Roscioni et al (2013) [62]	Case–control	88 T2DM	3	Urine	Progression of albuminuria stage	CE-MS	Yes
Merchant et al (2013) [74]	Case–control	16 + 17 T1DM	8–12	Urine	Renal functional decline of ≥3.3% per year	LC-MALDI-TOF-MS	No
Bhensdadia et al (2013) [60]	Post hoc analysis of RCT	204 T2DM	4	Urine	Renal functional decline	LC-MS/MS	No
Looker et al (2015) [76]	Nested case–control	154 + 153 T2DM	3.5	Serum	Loss of >40% of baseline eGFR during follow-up	LC-ESI-MS/MS	No
Pena et al (2015) [75]	Case–control	125 HT 88 T2DM	4	Plasma	Progression of albuminuria stage	LC-ESI-trap MS	No

Due to inconsistencies in methodology on how performance of the biomarker panels were assessed (e.g. testing the biomarker panels on top of clinical predictors [albuminuria and/or eGFR, etc.] vs testing the biomarker panel without clinical predictors), we do not report performance measures (e.g. AUC for receiver operating characteristic)

ESI, electrospray; iTRAQ, isobaric tags for relative and absolute quantification; MALDI, matrix-assisted laser desorption/ionisation; RCT, randomised controlled trial; T1DM, type 1 diabetes; T2DM, type 2 diabetes; TOF, time-of-flight

**Fig. 2:**
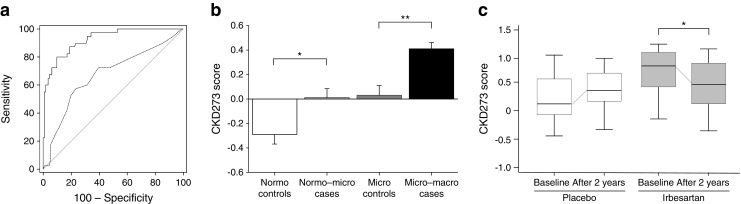
Overview of the CKD273 score for baseline risk prediction and drug response prediction. (**a**) Predictive ability of the CKD273 score in patients with diabetes and normoalbuminuria (*n* = 35 with 150 urine samples) at the time of urine sample collection up to 5 years prior to onset of diabetic nephropathy. The solid line shows the receiver operating characteristic (ROC) curve of the CKD273 score and the dashed line the ROC curve of the urinary albumin excretion rate (UAER) (*p* < 0.001 for difference in ROC curve between CKD273 score and UAER). Figure adapted from Zürbig et al [61]. (**b**) CKD273 score in patients with type 2 diabetes and normoalbuminuria (*n* = 48) or microalbuminuria (*n* = 40) at baseline. Patients transitioned during the albuminuria stage, whereas controls did not transition during follow-up. *p < 0.05, **p < 0.01 cases vs controls. White box, controls with normoalbuminuria; light grey box, patients with normo- to microalbuminuria; dark grey box, controls with microalbuminuria; black box, patients with micro- to macroalbuminuria. Figure adapted with permission from Roscioni et al [62]. (**c**) Box-and-whisker plots of the CKD273 score of patients from a nested case–control study in the IRMA-2 trial (*n* = 22) before (visit 2) and after 2 years (visit 9) of treatment with placebo (white boxes) or 300 mg irbesartan (grey boxes). *p < 0.05 baseline vs after 2 years. Figure adapted from Andersen et al [88]

The CKD273 score, a capillary electrophoresis–mass spectrometry (CE-MS)-based urinary peptide classifier, has been subsequently validated in several cohorts, many of them including patients with diabetes (Fig. 2). In a prospective study of 35 patients with type 1 or type 2 diabetes, the CKD273 score predicted subsequent progression to macroalbuminuria on average 5 years prior to its onset (Fig. 2a) [61]. Additionally, in a case–control study of 88 patients with type 2 diabetes, the CKD273 score predicted development of micro- or macroalbuminuria independently of any other renal risk marker (Fig. 2b) [62]. The discriminative ability of the CKD273 score for diabetic nephropathy was confirmed in a large, longitudinal multicentre study in which the CKD273 score improved prediction of accelerated eGFR decline on top of albuminuria and baseline eGFR [63]. Furthermore, analyses of both the Effect of Candesartan on Progression of Retinopathy in Type 1 Diabetes (DIRECT-Protect 1) and in Type 2 Diabetes (DIRECT-Protect 2) studies indicated that the CKD273 score improved risk prediction of the development of microalbuminuria independently of treatment and other physical or biochemical markers [64].

Although the above-mentioned studies validated the CKD273 score in external cohorts, these studies did not involve hard renal outcomes. In addition to external validation, one should also determine whether the predictive ability of a novel protein marker is sufficient to warrant a change in therapy. Whether starting treatment in a high-risk population identified by the CKD273 score will prevent microalbuminuria in type 2 diabetes is currently being investigated in the Proteomic Prediction and Renin–Angiotensin–Aldosterone System Inhibition Prevention of Early Diabetic Nephropathy in Type 2 Diabetic Patients With Normoalbuminuria trial (PRIORITY; ClinicalTrials.gov NCT02040441) [65]. This study does not involve hard renal outcomes (which would require extending the observation period for approximately 10 years) but specifically focuses on early detection of kidney disease using a surrogate endpoint of transition in albuminuria stage from normo- to microalbuminuria. The primary objective of the PRIORITY trial is to confirm that urinary proteomics can predict development of microalbuminuria. The PRIORITY trial will also assess whether high-risk patients identified by the CKD273 score will benefit from spironolactone therapy.

Results from these urinary proteomic studies have expanded our pathophysiological knowledge of DKD. Collagen fragments, especially those of the α1 type I collagen chain, have been shown to be significantly altered in urine 3–5 years before the onset of macroalbuminuria [61]. Several fragments of type I and type III collagen have been found in lower concentrations in patients with increased albuminuria levels, and positively correlate with a decline in eGFR [62]. Type I and type III α1 collagen and α2-HS-glycoprotein, among other peptides, were found to be prominent markers in a large cross-sectional multicentre study [65]. It is speculated that collagen fragments most likely originate from the kidney [61], and a decrease in collagen fragments in the urine of diabetic patients has been associated with the accumulation of extracellular matrix and increased fibrosis [66]. Distinguishing whether or not these peptides are specific to DKD or are reflective of age-related progression of renal functional decline in the non-diabetic population is important, as identifying specific DKD-related processes can lead to new therapeutic targets. Interestingly, in a study conducted in healthy individuals ranging in age from 2 to 73 years (*n* = 324), 49 age-related modifications of secretion in a number of peptides were observed. Of note, the downregulation of collagen fragments was especially evident [51]. Furthermore, these results were recently verified in a study involving 11,660 individuals [67]. Many of these peptides were previously described as biomarkers of CKD, likely indicating the well-known association between ageing and renal functional decline.

### Blood-derived proteomics

Proteomics in blood products is difficult to perform due to post-sampling variability and many high-abundance proteins (e.g. immunoglobulin, albumin) that can mask the low-abundant, potential biomarkers [68]. Additionally, the choice of anticoagulant agent used for plasma preparations, or the lack of an anticoagulant agent for serum collection, as well as the presence or absence of protease inhibitors, affects the activity of proteolytic enzymes in these samples [69]. Accordingly, early serum and plasma proteomic studies did not show much promise for biomarker discovery. On the one hand, one could argue that plasma proteomics offers an advantage over urine, as the number of substances is higher, and so there is a higher likelihood of detecting yet unknown biomarkers of kidney disease. On the other hand, circulating peptides could reflect general processes that are not specifically confined to the kidney. However, advancements in proteomics are pushing the field forward. Recent plasma proteomic studies have revealed new findings on the role of certain proteins in predicting kidney disease progression.

In a series of cross-sectional studies conducted in Denmark, plasma proteomic studies in patients with type 1 diabetes and nephropathy revealed several candidate proteins, including C3f and apolipoprotein C-I, apolipoprotein A-I, transthyretin and cystatin C [70–72]. In a recent cross-sectional study, untargeted plasma proteome analysis revealed significant differences in more than 300 proteins in patients with early-stage CKD compared with patients receiving haemodialysis [73]. Preliminary validation experiments demonstrated that one of these proteins, leucine-rich α2-glycoprotein, was significantly associated with higher mortality in CKD stage 5 [73]. Investigation of the predictive value of several of the identified potential biomarkers in a larger cohort is currently ongoing, using targeted MS.

Small, prospective studies have yielded further insights in the use of plasma proteomics in studying DKD (Table 2) [74, 75]. A large study combining hypothesis-driven (ELISA, Luminex [Austin, TX, USA]) and hypothesis-free (liquid chromatography–mass spectrometry [LC-MS] platforms) approaches in a nested case–control design (*n* = 307) was recently performed [76]. This study measured 207 serum biomarkers and identified 35 that were significantly associated with rapid progression of eGFR decline. Furthermore, a sparser set of 14 biomarkers contained most of the predictive information beyond clinical covariates. Novel biomarkers identified included FGF-21, the symmetric to asymmetric dimethylarginine ratio (SDMA/ADMA), β2-microglobulin, C16-acylcarnitine, kidney injury molecule-1 and uracil [76]. In another large study, targeted MS was performed to predict progression of DKD in 279 patients (healthy controls, patients with mild diabetic nephropathy and patients with severe diabetic nephropathy) during discovery and validation phases [77]. During the discovery phase, 150–200 proteins were identified from over 155,000 MS/MS spectra. A total of 275 proteins were identified and quantified. Of these, >50 proteins showed statistically significant differences between disease states. Eventually, a panel of 13 biomarkers was developed into the targeted MS assay PromarkerD (Proteomics International, Perth, WA, Australia) [78]. Proteins involved in inflammation, metabolism and oxidative stress are included in the panel. The two aforementioned studies examined sparser protein panels based on the strongest protein predictors instead of all proteins showing a statistically significant association. Future studies will help determine the clinical utility of smaller, well-defined panels of predictive biomarkers identified through proteomics and developed into easy-to-use assays or point-of-care analysers for use in clinical practice.

Furthermore, to overcome issues of blood-based proteomics, a novel targeted proteomic technique based on aptamer technology has been introduced (SOMAscan assay; SomaLogic, Boulder, CO, USA) [79]. This proteomic assay can currently measure 1310 protein analytes in serum, plasma or cerebrospinal fluid. This assay measures native proteins in complex matrices by transforming each individual protein concentration into a corresponding SOMAmer reagent concentration, which is then quantified by standard techniques such as microarrays or quantitative real-time PCR. The approach has been used to identify 58 potential CKD biomarkers [80]. Unfortunately, however, validation of the results presented 5 years ago is still pending. Work assessing associations of plasma proteins measured with this technology is currently ongoing in patients with type 1 diabetes [81].

## Proteomics and response to therapy

At least as important as risk prediction is the quest to find proteins predicting how an individual responds to treatment. Many of the studies predicting disease progression do not provide any information on how an individual would respond to a drug, or identify patients who are prone to side effects [82, 83]. Yet, individual patients show a wide variability in the way they respond to drugs [84]. From a clinical decision perspective, being able to predict a good response to a specific intervention would be time and cost saving and would be ultimately beneficial for the individual patient. Currently in clinical practice, there is no way to predict a patient’s response to renoprotective therapy. A physician’s best tool is to tailor medication with respect to a patient’s specific clinical presentation [85]. Proteomics could offer a solution to improve tailoring of medication to those more likely to respond or less likely to experience side effects. First, the individual proteome can be used to phenotype a patient with a good or poor therapy response before treatment is initiated: a so-called ‘baseline risk prediction’. Another strategy is to monitor changes in the proteome after a few weeks’ therapy to select individuals more likely to benefit from subsequent clinical outcomes, a so-called ‘dynamic response prediction’. Studies evaluating the predictive value of baseline proteins or changes in proteins during the first week of therapy are therefore an area of interest for the future. Such studies would also generate information about novel drug targets, as changes in the proteome during medication exposure will provide insight into molecular mechanisms and processes of drug effects [86].

Proteomic studies for baseline and dynamic drug response prediction in diabetes are still in their infancy and only a few studies have been conducted. One study investigated the ability of the aforementioned CKD273 score to predict the response to spironolactone therapy in patients with type 2 diabetes and therapy-resistant hypertension. The study suggested that patients in the upper tertile of the CKD273 score were more likely to show an albuminuria-lowering response (−64% [95% CI −84%, −20%] placebo-corrected reduction) to spironolactone compared with patients in the lower tertile of the CKD score (−13% [95% CI −52%, +59%] albuminuria reduction relative to placebo) (M. Lindhardt, F. Persson, C. Oxlund, I. A. Jacobsen, P. Zürbig, H. Mischak, P. Rossing, H. J. L. Heerspink, unpublished observations). With respect to baseline response prediction in other omics platforms, a serum metabolomics study for prediction of therapeutic response to spironolactone therapy in diabetes has been conducted. A panel of 21 metabolites improved prediction of albuminuria response on top of clinical variables [87]. These studies are a first attempt to use omics-based approaches for development of drug response classifiers. Further studies in large datasets are required to validate and implement these studies in clinical practice.

Assessing drug response by investigating proteomic changes may also give further insights into the mechanisms of drug response. The change in the proteome may be used as an indicator of subsequent drug efficacy on clinical outcomes. Changes in urinary peptides were observed after treatment with varying doses of candesartan in a randomised double-blind crossover trial. Fifteen of 113 polypeptides in patients with macroalbuminuria were significantly changed after treatment, towards levels of patients with normoalbuminuria [66]. Furthermore, changes in the urinary proteome were observed in a nested case–control study of a clinical trial in hypertensive type 2 diabetic patients with microalbuminuria randomised for treatment with irbesartan or placebo. Significant changes in the urinary CKD273 score were observed after 2 years’ follow-up in the irbesartan group, but not in the placebo group (Fig. 2c) [88]. However, it should be noted that in the latter study, changes in the proteome were only measured 2 years after follow-up. In clinical practice, drug efficacy is usually monitored after a few weeks of therapy. It is currently unknown whether changes in the proteome are already present during the first weeks of treatment with RAAS intervention. In this respect, it is of interest to note that changes in the urine proteome after short-term dietary intervention with olive oil [89] and in the plasma proteome after short-term treatment with a low-energy diet have been observed [90], implying that proteomics can indeed be used as a therapy-monitoring tool.

## Recommendations for the future

Before a biomarker or panel of biomarkers can be used in clinical practice, it needs to be extensively validated in order to assess its effect on patient management and outcomes. The translation of a proteomic panel from discovery to clinical practice is a process full of pitfalls and limitations. A framework for the development of biomarkers has been proposed [91]. While a number of candidate proteomic biomarkers have been identified that can predict progression of DKD, many biomarker studies are limited by small sample sizes, heterogeneity of results, and a lack of large validation studies. As a result, novel biomarkers do not proceed past the initial discovery phase and do not progress further down the biomarker pipeline into validation and clinical utility stages [92]. To date, no MS-based in vitro diagnostic device for the measurement of proteins and peptides has been cleared or approved by the Food and Drug Administration for marketing or use in clinical trials in DKD [93]. More awareness and investments need to be made to perform well-powered studies appropriately addressing the clinical utility and clinical outcome phases in order to start implementing proteomic biomarkers in clinical practice. To conduct such large validation studies, collaborations between academia and industry may be a strategy to share expertise from different areas and to promote effective dissemination of results.

A review of the literature clearly indicates that multiple biomarker candidates are available, and the data demonstrating significant association with DKD are well developed. However, the clinical utility of these biomarker candidates has generally not been addressed. To enable true advancement (and implementation of the biomarkers), appropriate study designs need to be developed, ideally jointly with the regulatory agency. As pointed out previously: as long as a hard endpoint is a mandatory requirement, implementation of biomarkers will be blocked. Furthermore, the study of proteomics is only one aspect of the entire system. Concomitant deep investigation and integration of complex datasets of the genome, transcriptome, proteome and metabolome will give a more detailed understanding of the pathophysiology of DKD [94].

To enable progress, we also need to shift the focus from discovery towards validation. Multiple biomarkers should be assessed in a comparative way for their diagnostic or prognostic value, ideally in the same samples. Similar approaches have been advocated and are now implemented in other fields, such as bladder cancer [95]. Such a combined approach would also enable combining multiple biomarkers into a classifier fit for any specific purpose and would enable a path forwards towards personalised medicine.

From a technical point of view, we anticipate that, in the future, immunological assays will be replaced by targeted MS, employing multiple reaction monitoring (MRM) [96]. In several recent publications, MRM-based assays were found to be equal or superior to antibody-based approaches [97]. Also, the ability for multiplexing renders this approach very promising. In combination with higher sensitivity and better selectivity in comparison with antibodies, we may see that MRM-based assays will be used in the future for routine targeted proteome analysis.

Another promising approach is the use of CE-MS in an untargeted analysis to assess multiple diseases. This approach could be used to assess the complete urinary peptidome (limited by the detection limit, but typically analysing several thousand peptides), and then specifically address the risk of developing CKD, chronic heart failure, cardiovascular disease, etc., based on specific biomarker signatures. Owing to the high complexity and the aim to assess multiple diseases, a targeted proteomic approach for this purpose (e.g. based on MRM) does not seem possible.

Irrespective of the diagnostic value of proteomic biomarkers, changes in the proteome also provide information about disease pathophysiology, and will enable ways to identify more appropriate therapeutic targets. As outlined in detail in recent reviews [86, 98], application of this strategy holds the promise to identify the actual molecular cause of DKD, and consequently enable targeted intervention.

## Conclusions

Despite stringent blood glucose and blood pressure control with RAAS inhibitors, the incidence of RRT continues to grow. Early identification of individuals at risk of progressive loss of renal function and administration of appropriate treatment will delay progression to RRT. The measurement of the proteome offers an opportunity for early identification of individuals at risk of further disease progression. However, to assess efficacy early in the course of DKD, a change is required in the use of clinical efficacy endpoints. The current endpoints—ESRD, doubling of serum creatinine or halving of kidney function—typically take 10–20 years to develop. Clinical trials in the early stages of disease using these endpoints are practically impossible. Alternative endpoints need to be accepted in order to foster drug efficacy assessment in the early stages of disease. The European Medicines Agency proposed endpoint of CKD stage 3 in intervention trials [15] is an encouraging sign that early intervention strategies can be developed.

Given the large heterogeneity in the pathophysiology of DKD, a panel of proteins/peptides capturing the various disease progression pathways is more likely to predict disease progression or response to therapy than a single protein. Predicting the risk of an individual and directing drugs to those at highest risk is not sufficient to optimise therapy, as it is unlikely that all high-risk individuals will also respond to treatment. Therefore, additional studies are needed to validate proteomic biomarkers in order to identify individuals more likely to respond favourably to treatment. It will be a high priority on research agendas to tailor therapy and minimise side effects, thereby potentially reducing the burden of DKD.

## Abbreviations

CE-MS: Capillary electrophoresis–mass spectrometry
CKD: Chronic kidney disease
DKD: Diabetic kidney disease
eGFR: Estimated glomerular filtration rate
ESRD: End-stage renal disease
FGF: Fibroblast growth factor
IRMA-2: Irbesartan Microalbuminuria Study-2
LC-MS: Liquid chromatography–mass spectrometry
MRM: Multiple reaction monitoring
MS: Mass spectrometry
PRIORITY trial: Proteomic Prediction and Renin–Angiotensin–Aldosterone System Inhibition Prevention of Early Diabetic Nephropathy in Type 2 Diabetic Patients With Normoalbuminuria trial
RAAS: Renin–angiotensin–aldosterone system
RRT: Renal replacement therapy
SELDI: Surface-enhanced laser desorption ionisation
TNFR: Tumour necrosis factor receptor
UAE: Urinary albumin excretion
VEGF: Vascular endothelial growth factor

## References

[1] Afkarian M, Sachs MC, Kestenbaum B (2013). Kidney disease and increased mortality risk in type 2 diabetes. J Am Soc Nephrol.

[2] United States Renal Data System (2015) USRDS annual data report: epidemiology of kidney disease in the United States. National Institutes of Health, National Institute of Diabetes and Digestive and Kidney Diseases, Bethesda, MD

[3] Pippias M, Jager KJ, Kramer A et al (2016) The changing trends and outcomes in renal replacement therapy: data from the ERA-EDTA Registry. Nephrol Dial Transplant 31:831–841 10.1093/ndt/gfv327 26361801

[4] Liyanage T, Ninomiya T, Jha V (2015). Worldwide access to treatment for end-stage kidney disease: a systematic review. Lancet.

[5] National Kidney Foundation (2012) KDOQI clinical practice guideline for diabetes and CKD: 2012 update. Am J Kidney Dis 60:850–886 10.1053/j.ajkd.2012.07.005 23067652

[6] Heerspink HJL, Ninomiya T, Persson F (2016). Is a reduction in albuminuria associated with renal and cardiovascular protection? A post-hoc analysis of the ALTITUDE trial. Diabetes Obes Metab.

[7] De Zeeuw D, Remuzzi G, Parving HH (2004). Proteinuria, a target for renoprotection in patients with type 2 diabetic nephropathy: lessons from RENAAL. Kidney Int.

[8] de Boer IH, Afkarian M, Rue TC (2014). Renal outcomes in patients with type 1 diabetes and macroalbuminuria. J Am Soc Nephrol.

[9] Viazzi F, Leoncini G, Conti N (2010). Microalbuminuria is a predictor of chronic renal insufficiency in patients without diabetes and with hypertension: the MAGIC study. Clin J Am Soc Nephrol.

[10] Ninomiya T, Perkovic V, de Galan BE (2009). Albuminuria and kidney function independently predict cardiovascular and renal outcomes in diabetes. J Am Soc Nephrol.

[11] Susztak K, Bottinger EP (2006). Diabetic nephropathy: a frontier for personalized medicine. J Am Soc Nephrol.

[12] Schievink B, Kropelin T, Mulder S (2016). Early renin-angiotensin system intervention is more beneficial than late intervention in delaying end-stage renal disease in patients with type 2 diabetes. Diabetes Obes Metab.

[13] Inker LA, Lambers Heerspink HJ, Mondal H (2014). GFR decline as an alternative end point to kidney failure in clinical trials: a meta-analysis of treatment effects from 37 randomized trials. Am J Kidney Dis.

[14] Roscioni SS, Lambers Heerspink HJ, de Zeeuw D (2014). Microalbuminuria: target for renoprotective therapy PRO. Kidney Int.

[15] European Medicines Agency (2014) Guideline on the clinical investigation of medicinal products to prevent development/slow progression of chronic renal insufficiency. Available from http://www.ema.europa.eu/docs/en_GB/document_library/Scientific_guideline/2014/06/WC500169469.pdf, accessed 15 March 2016

[16] Niewczas MA, Gohda T, Skupien J (2012). Circulating TNF receptors 1 and 2 predict ESRD in type 2 diabetes. J Am Soc Nephrol.

[17] Gohda T, Niewczas MA, Ficociello LH (2012). Circulating TNF receptors 1 and 2 predict stage 3 CKD in type 1 diabetes. J Am Soc Nephrol.

[18] Mischak H, Delles C, Vlahou A, Vanholder R (2015). Proteomic biomarkers in kidney disease: issues in development and implementation. Nat Rev Nephrol.

[19] Tam FW, Riser BL, Meeran K, Rambow J, Pusey CD, Frankel AH (2009). Urinary monocyte chemoattractant protein-1 (MCP-1) and connective tissue growth factor (CCN2) as prognostic markers for progression of diabetic nephropathy. Cytokine.

[20] Persson F, Rathcke CN, Gall MA, Parving HH, Vestergaard H, Rossing P (2012). High YKL-40 levels predict mortality in patients with type 2 diabetes. Diabetes Res Clin Pract.

[21] Hellemons ME, Mazagova M, Gansevoort RT (2012). Growth-differentiation factor 15 predicts worsening of albuminuria in patients with type 2 diabetes. Diabetes Care.

[22] Conway BR, Manoharan D, Manoharan D (2012). Measuring urinary tubular biomarkers in type 2 diabetes does not add prognostic value beyond established risk factors. Kidney Int.

[23] Fufaa GD, Weil EJ, Nelson RG (2015). Association of urinary KIM-1, L-FABP, NAG and NGAL with incident end-stage renal disease and mortality in American Indians with type 2 diabetes mellitus. Diabetologia.

[24] Jorsal A, Tarnow L, Frystyk J (2008). Serum adiponectin predicts all-cause mortality and end stage renal disease in patients with type I diabetes and diabetic nephropathy. Kidney Int.

[25] Titan SM, Zatz R, Graciolli FG (2011). FGF-23 as a predictor of renal outcome in diabetic nephropathy. Clin J Am Soc Nephrol.

[26] Hovind P, Rossing P, Tarnow L, Johnson RJ, Parving HH (2009). Serum uric acid as a predictor for development of diabetic nephropathy in type 1 diabetes: an inception cohort study. Diabetes.

[27] Ficociello LH, Rosolowsky ET, Niewczas MA (2010). High-normal serum uric acid increases risk of early progressive renal function loss in type 1 diabetes: results of a 6-year follow-up. Diabetes Care.

[28] Panduru NM, Forsblom C, Saraheimo M (2013). Urinary liver-type fatty acid-binding protein and progression of diabetic nephropathy in type 1 diabetes. Diabetes Care.

[29] Morton J, Zoungas S, Li Q (2012). Low HDL cholesterol and the risk of diabetic nephropathy and retinopathy: results of the ADVANCE study. Diabetes Care.

[30] Ju W, Nair V, Smith S (2015). Tissue transcriptome-driven identification of epidermal growth factor as a chronic kidney disease biomarker. Sci Transl Med.

[31] Fechete R, Heinzel A, Perco P (2011). Mapping of molecular pathways, biomarkers and drug targets for diabetic nephropathy. Proteomics Clin Appl.

[32] Pena MJ, de Zeeuw D, Mischak H (2015). Prognostic clinical and molecular biomarkers of renal disease in type 2 diabetes. Nephrol Dial Transplant.

[33] Persson F, Rossing P, Hovind P (2008). Endothelial dysfunction and inflammation predict development of diabetic nephropathy in the Irbesartan in Patients with Type 2 Diabetes and Microalbuminuria (IRMA 2) study. Scand J Clin Lab Invest.

[34] Astrup AS, Tarnow L, Pietraszek L (2008). Markers of endothelial dysfunction and inflammation in type 1 diabetic patients with or without diabetic nephropathy followed for 10 years: association with mortality and decline of glomerular filtration rate. Diabetes Care.

[35] Verhave JC, Bouchard J, Goupil R (2013). Clinical value of inflammatory urinary biomarkers in overt diabetic nephropathy: a prospective study. Diabetes Res Clin Pract.

[36] Agarwal R, Duffin KL, Laska DA, Voelker JR, Breyer MD, Mitchell PG (2014). A prospective study of multiple protein biomarkers to predict progression in diabetic chronic kidney disease. Nephrol Dial Transplant.

[37] Pena MJ, Heinzel A, Heinze G (2015). A panel of novel biomarkers representing different disease pathways improves prediction of renal function decline in type 2 diabetes. PLoS One.

[38] Avogaro A, Fadini GP, Gallo A, Pagnin E, de Kreutzenberg S (2006). Endothelial dysfunction in type 2 diabetes mellitus. Nutr Metab Cardiovasc Dis.

[39] Rabelink TJ, de Zeeuw D (2015). The glycocalyx-linking albuminuria with renal and cardiovascular disease. Nat Rev Nephrol.

[40] Broekhuizen LN, Lemkes BA, Mooij HL (2010). Effect of sulodexide on endothelial glycocalyx and vascular permeability in patients with type 2 diabetes mellitus. Diabetologia.

[41] Garsen M, Rops AL, Rabelink TJ, Berden JH, van der Vlag J (2014). The role of heparanase and the endothelial glycocalyx in the development of proteinuria. Nephrol Dial Transplant.

[42] Banks RE, Dunn MJ, Hochstrasser DF (2000). Proteomics: new perspectives, new biomedical opportunities. Lancet.

[43] Stein LD (2004). Human genome: end of the beginning. Nature.

[44] Dihazi H, Muller GA, Lindner S (2007). Characterization of diabetic nephropathy by urinary proteomic analysis: identification of a processed ubiquitin form as a differentially excreted protein in diabetic nephropathy patients. Clin Chem.

[45] Papale M, Di Paolo S, Magistroni R (2010). Urine proteome analysis may allow noninvasive differential diagnosis of diabetic nephropathy. Diabetes Care.

[46] Papale M, Di Paolo S, Vocino G, Rocchetti MT, Gesualdo L (2014). Proteomics and diabetic nephropathy: what have we learned from a decade of clinical proteomics studies?. J Nephrol.

[47] Mischak H, Ioannidis JP, Argiles A (2012). Implementation of proteomic biomarkers: making it work. Eur J Clin Investig.

[48] Merchant ML (2010). Mass spectrometry in chronic kidney disease research. Adv Chronic Kidney Dis.

[49] Merchant ML, Perkins BA, Boratyn GM (2009). Urinary peptidome may predict renal function decline in type 1 diabetes and microalbuminuria. J Am Soc Nephrol.

[50] Good DM, Zürbig P, Argiles A (2010). Naturally occurring human urinary peptides for use in diagnosis of chronic kidney disease. Mol Cell Proteomics.

[51] Zürbig P, Decramer S, Dakna M (2009). The human urinary proteome reveals high similarity between kidney aging and chronic kidney disease. Proteomics.

[52] Gansevoort RT, Ritz E (2009). Hermann Senator and albuminuria—forgotten pioneering work in the 19th century. Nephrol Dial Transplant.

[53] Mischak H (2015). Pro: urine proteomics as a liquid kidney biopsy: no more kidney punctures!. Nephrol Dial Transplant.

[54] Thongboonkerd V, Malasit P (2005). Renal and urinary proteomics: current applications and challenges. Proteomics.

[55] Ben Ameur R, Molina L, Bolvin C (2010). Proteomic approaches for discovering biomarkers of diabetic nephropathy. Nephrol Dial Transplant.

[56] Jankowski J, Schanstra JP, Mischak H (2015). Body fluid peptide and protein signatures in diabetic kidney diseases. Nephrol Dial Transplant.

[57] Critselis E, Lambers Heerspink H (2016) Utility of the CKD273 peptide classifier in predicting chronic kidney disease progression. Nephrol Dial Transplant 31:249–254 10.1093/ndt/gfv062 25791724

[58] Otu HH, Can H, Spentzos D (2007). Prediction of diabetic nephropathy using urine proteomic profiling 10 years prior to development of nephropathy. Diabetes Care.

[59] Schlatzer D, Maahs DM, Chance MR (2012). Novel urinary protein biomarkers predicting the development of microalbuminuria and renal function decline in type 1 diabetes. Diabetes Care.

[60] Bhensdadia NM, Hunt KJ, Lopes-Virella MF (2013). Urine haptoglobin levels predict early renal functional decline in patients with type 2 diabetes. Kidney Int.

[61] Zürbig P, Jerums G, Hovind P (2012). Urinary proteomics for early diagnosis in diabetic nephropathy. Diabetes.

[62] Roscioni SS, de Zeeuw D, Hellemons ME (2013). A urinary peptide biomarker set predicts worsening of albuminuria in type 2 diabetes mellitus. Diabetologia.

[63] Schanstra JP, Zürbig P, Alkhalaf A (2015). Diagnosis and prediction of CKD progression by assessment of urinary peptides. J Am Soc Nephrol.

[64] Lindhardt M, Persson F, Zürbig P et al (2016) Urinary proteomics predict onset of microalbminuria in normoalbuminuric type 2 diabetic patients, a sub-study of the DIRECT-Protect 2 study. Nephrol Dial Transpl (in press) 10.1093/ndt/gfw292 27507891

[65] Siwy J, Schanstra JP, Argiles A (2014). Multicentre prospective validation of a urinary peptidome-based classifier for the diagnosis of type 2 diabetic nephropathy. Nephrol Dial Transplant.

[66] Rossing K, Mischak H, Parving HH (2005). Impact of diabetic nephropathy and angiotensin II receptor blockade on urinary polypeptide patterns. Kidney Int.

[67] Nkuipou-Kenfack E, Bhat A, Klein J (2015). Identification of ageing-associated naturally occurring peptides in human urine. Oncotarget.

[68] Kolch W, Neususs C, Pelzing M, Mischak H (2005). Capillary electrophoresis-mass spectrometry as a powerful tool in clinical diagnosis and biomarker discovery. Mass Spectrom Rev.

[69] Jambunathan K, Galande AK (2014). Sample collection in clinical proteomics—proteolytic activity profile of serum and plasma. Proteomics Clin Appl.

[70] Overgaard AJ, Hansen HG, Lajer M (2010). Plasma proteome analysis of patients with type 1 diabetes with diabetic nephropathy. Proteome Sci.

[71] Overgaard AJ, Thingholm TE, Larsen MR (2010). Quantitative iTRAQ-based proteomic identification of candidate biomarkers for diabetic nephropathy in plasma of type 1 diabetic patients. Clin Proteomics.

[72] Hansen HG, Overgaard J, Lajer M (2010). Finding diabetic nephropathy biomarkers in the plasma peptidome by high-throughput magnetic bead processing and MALDI-TOF-MS analysis. Proteomics Clin Appl.

[73] Glorieux G, Mullen W, Duranton F (2015). New insights in molecular mechanisms involved in chronic kidney disease using high-resolution plasma proteome analysis. Nephrol Dial Transplant.

[74] Merchant ML, Niewczas MA, Ficociello LH (2013). Plasma kininogen and kininogen fragments are biomarkers of progressive renal decline in type 1 diabetes. Kidney Int.

[75] Pena MJ, Jankowski J, Heinze G (2015). Plasma proteomics classifiers improve risk prediction for renal disease in patients with hypertension or type 2 diabetes. J Hypertens.

[76] Looker HC, Colombo M, Hess S (2015). Biomarkers of rapid chronic kidney disease progression in type 2 diabetes. Kidney Int.

[77] Lipscombe R (2015). Translating biomarker discovery into a diagnostic test for diabetic kidney disease. Presented at the 11th Australian Peptide Conference 2015, Kingscliff, NSW, Australia. Available from http://www.ozpeptide.org/australian-peptide-conference-2015/program, accessed 15 March 2016 (Abstract)

[78] Bringans S, Casey T, Davis T et al (2012) Biomarkers associated with pre-diabetes, diabetes and diabetes related conditions. Patent Application PCT/AU2011/001212

[79] Lollo B, Steele F, Gold L (2014). Beyond antibodies: new affinity reagents to unlock the proteome. Proteomics.

[80] Gold L, Ayers D, Bertino J (2010). Aptamer-based multiplexed proteomic technology for biomarker discovery. PLoS One.

[81] Schlafly A, Niewczas M, Pezzolesi M, Krolewski A (2015). Plasma proteins associated with declining renal function in patients with type 1 diabetes: results of a global proteomic analysis using SOMAscan platform. J Am Soc Nephrol.

[82] de Zeeuw D, Akizawa T, Audhya P (2013). Bardoxolone methyl in type 2 diabetes and stage 4 chronic kidney disease. N Engl J Med.

[83] Brenner BM, Cooper ME, de Zeeuw D (2001). Effects of losartan on renal and cardiovascular outcomes in patients with type 2 diabetes and nephropathy. N Engl J Med.

[84] Schievink B, de Zeeuw D, Parving HH, Rossing P, Lambers Heerspink HJ (2015). The renal protective effect of angiotensin receptor blockers depends on intra-individual response variation in multiple risk markers. Br J Clin Pharmacol.

[85] Klonoff DC (2015). Precision medicine for managing diabetes. J Diabetes Sci Technol.

[86] Lambers Heerspink HJ, Oberbauer R, Perco P (2015). Drugs meeting the molecular basis of diabetic kidney disease: bridging from molecular mechanism to personalized medicine. Nephrol Dial Transplant.

[87] Pena M, Mayer B, Heinzel A, Rossing P, Lambers Heerspink HJ (2015). A serum metabolite classifier predicts response to ARBs in diabetes. J Am Soc Nephrol.

[88] Andersen S, Mischak H, Zürbig P, Parving HH, Rossing P (2010). Urinary proteome analysis enables assessment of renoprotective treatment in type 2 diabetic patients with microalbuminuria. BMC Nephrol.

[89] Silva S, Bronze MR, Figueira ME (2015). Impact of a 6-wk olive oil supplementation in healthy adults on urinary proteomic biomarkers of coronary artery disease, chronic kidney disease, and diabetes (types 1 and 2): a randomized, parallel, controlled, double-blind study. Am J Clin Nutr.

[90] Sleddering MA, Markvoort AJ, Dharuri HK (2014). Proteomic analysis in type 2 diabetes patients before and after a very low calorie diet reveals potential disease state and intervention specific biomarkers. PLoS One.

[91] Hlatky MA, Greenland P, Arnett DK (2009). Criteria for evaluation of novel markers of cardiovascular risk: a scientific statement from the American Heart Association. Circulation.

[92] Schutte E, Gansevoort RT, Benner J, Lutgers HL, Lambers Heerspink HJ (2015). Will the future lie in multitude? A critical appraisal of biomarker panel studies on prediction of diabetic kidney disease progression. Nephrol Dial Transplant.

[93] Lathrop JT, Jeffery DA, Shea YR, Scholl PF, Chan MM (2016). US Food and Drug Administration perspectives on clinical mass spectrometry. Clin Chem.

[94] Conserva F, Gesualdo L, Papale M (2016). A systems biology overview on human diabetic nephropathy: from genetic susceptibility to post-transcriptional and post-translational modifications. J Diabetes Res.

[95] Vlahou A (2011). Back to the future in bladder cancer research. Expert Rev Proteomics.

[96] Domanski D, Percy AJ, Yang J (2012). MRM-based multiplexed quantitation of 67 putative cardiovascular disease biomarkers in human plasma. Proteomics.

[97] Mermelekas G, Vlahou A, Zoidakis J (2015). SRM/MRM targeted proteomics as a tool for biomarker validation and absolute quantification in human urine. Expert Rev Mol Diagn.

[98] Cisek K, Krochmal M, Klein J, Mischak H (2015) The application of multi-omics and systems biology to identify therapeutic targets in chronic kidney disease. Nephrol Dial Transplant doi:10.1093/ndt/gfv364 10.1093/ndt/gfv364 26487673

